# Supplementary material for: Are carbon emissions trading and green financial instruments synergistic? -Comprehensive quantitative research based on content analysis
Source: PLoS One. 2024 Mar 7;19(3):e0298601. doi: 10.1371/journal.pone.0298601 (PMC10919601; doi:10.1371/journal.pone.0298601)
Supplement: S1 Data — (ZIP) [file pone.0298601.s001.zip › Data/地级市Z_F工作报告.zip!/╔╜╢1⁄2/╠⌐░▓╩╨/└┤╫╘╣1⁄2╓┌║┼í╛┬φ┐╦╩2╛▌═°í┐_2019.docx]

各位代表：

现在，我代表市人民政府向大会报告工作，请予审议，并请市政协委员和列席会议的同志提出意见。

一、2018年工作回顾

2018年，是全市上下凝心聚力、砥砺奋进的一年。在省委、省政府和市委坚强领导下，我们坚持以习近平新时代中国特色社会主义思想和党的十九大精神为指导，认真学习贯彻习近平总书记视察山东重要讲话、重要指示批示精神，坚持稳中求进工作总基调，在高质量发展道路上迈出新步伐。全市生产总值实现3651.5亿元、增长5.7%；完成一般公共预算收入219.5亿元、增长6%，其中税收收入158亿元、增长7.6%，税收占比72%、提高1.1个百分点；社会消费品零售总额1593.8亿元、增长8.8%；进出口总额160.8亿元，其中出口127.6亿元、增长8.1%；城镇、农村居民人均可支配收入分别增长7.5%和8.2%，连年保持平稳持续增长。

（一）产业发展取得新成效。坚定实施“产业兴市”战略，坚定不移支持民营经济发展，加大对重点产业、骨干企业培植力度，实体经济发展活力增强。全市规模以上工业增加值、主营业务收入、利润分别增长5.5%、7.4%、20.5%。泰山石膏、山能重装营业收入突破100亿元，全市过百亿元企业达到6家。以全域旅游为抓手，大力实施交通组织换乘、特色餐饮小吃、停车场提升、旅游厕所提升“四大工程”，市政中心东侧占地56亩、可提供710个停车位的生态停车场建成启用；泰山门票改为三天内有效；接待国内外游客人次、旅游消费总额分别增长10.1%和13.4%。现代物流加快发展，峰松物流、速恒物流、中国供销（宁阳）农副产品物流园等项目建成运营，山东宏禾物流公司被认定为国家级城市高效配送示范企业，岱岳经济开发区综合物流产业园荣获全国优秀物流园区称号。全市电商交易额达到1213亿元、增长26%，13个村被阿里巴巴评为中国“淘宝村”。深入实施文化产业“十百千”工程，省级以上文化产业示范基地累计达到12家，泰山新闻出版小镇等一批重点文化产业项目加快建设。高标准编制乡村振兴战略规划及配套方案，乡村振兴战略扎实推进；新泰市和全市5个乡镇、60个村列入全省乡村振兴“十百千”示范创建单位；新增高标准基本农田3.82万亩，粮食生产保持稳定；有机蔬菜、泰山茶、特色果品、高档苗木花卉种植面积分别达到30.2万亩、4.5万亩、171万亩、42.5万亩，农业龙头企业达到538家；奶牛存栏和奶产量连续12年保持全省首位，发展经验在全国推广；泰山区农高区成功入选国家现代农业产业园创建名单。

（二）新旧动能转换全面起势。着力构建新旧动能转换规划体系、政策配套体系、“六个一”工作推进体系，31个项目入选省新旧动能转换重大项目库第一批优选项目，233个市级新旧动能转换重大项目完成投资660亿元。加快推进泰安二期抽水蓄能电站、京杭运河东平湖湖区航道及作业区、正大新能源、复合材料产业研究院、泰山玻纤产业园、海天智能机器人、晋煤明升达洁净煤气化等一批重大项目。诺莱医养、康复辅具产业园等一批医养健康产业项目加快建设。14家企业上榜全省“瞪羚”企业名单。研究制定支持工业领军企业和科技创新型企业“双50强”发展意见，设立100亿元的重点企业财源建设基金，首批共扶持重点企业5家，落实资金12.8亿元；出台人才“金十条”政策，设立6亿元招才引智基金，年内兑现奖励扶持资金3022.6万元。投资14.9亿元，建成13.3万平方米的泰山创新谷和国家级高创中心孵化器，引进并注册公司团队49家。全市研发经费投入强度连续4年超过全省平均水平。农大肥业技术中心获批国家企业技术中心，全市国家级创新平台达到9家。2项发明专利获中国专利银奖，3项专利获优秀奖。净增高新技术企业57家，全年规模以上工业企业实现高新技术产业产值同比增长8.51%，占规模以上工业总产值的比重达到36.89%。新增中国驰名商标5件，全市名牌总量达到50件。石横特钢泰安建筑用钢产业集群被列为全省四大钢铁产业集群之一。康平纳集团“筒子纱智能染色工业示范项目”荣获第五届中国工业大奖。东平超大型粉煤气化工程示范项目列入国家级重点研发计划。峰松物流与北京汇通天下合作成立全国无车承运结算中心。

（三）改革开放力度不断加大。坚持把“放管服”改革作为破解发展环境难点、痛点、堵点的利器，在全省率先提出“贴心代办，一次办好”，共向县（市、区）下放市级行政权力4120项，委托功能区行使市级行政权力3676项。实行“六十二证合一”，整合数量位居全省首位。设立全省第一家市级行政审批服务局，被列入山东省庆祝改革开放40周年最具影响力事件。全年新登记市场主体6.2万户，增长10.9%。加大供给侧结构性改革力度，能源供给结构得到优化，可再生能源装机突破267万千瓦；全面落实国家减税降费政策，减免税费88.25亿元。农业农村改革进一步深化，全市基本完成清产核资工作，93%的村（居）完成集体产权改革。国资国企、财税金融、统计审计等领域改革深入实施。市县机构改革顺利推进。社会信用体系建设、公共资源交易制度改革取得新成效。

突出抓好“双招双引”，成功举办第三十二届泰山国际登山节暨2018中国泰安投资合作洽谈会，集中签约50个项目，总投资1720亿元；积极参与首届山东儒商大会、香港山东周等活动，取得丰硕成果。泰山Office、华能新能源新材料、中信重工开诚智能特种机器人、唯品会山东物流园、万吨级芳纶新材料产业园、隆基科技城、泰山云安智能产业园、泰山国际生态健康城、华润万象汇等一批大项目落地建设。全年实际利用外资46亿元、增长16.6%；在建招商引资项目到位市外资金373.4亿元、增长11%。引进“两院”院士3人、国家“千人计划”“万人计划”专家9人，省级以上高层次人才总量达到130人。组织企业走出去，积极参与“一带一路”建设，新增外贸获权企业279家，与180个国家和地区建立了贸易往来。扎实开展结对共建活动，加大对重庆巫溪县、新疆岳普湖县对口帮扶力度。

（四）山水生态城市建设扎实推进。科学编制《泰安市城乡一体空间发展战略规划》。泰城博阳路、万官大街、凤凰路等一批群众期盼已久的城市道路建成通车，擂鼓石大街中段、环山路东延改造工程顺利推进。坚持城市规划民生优先，把最好的地段留给市民，腾空市值7.5亿元、占地136亩的岱庙北侧土地，改造建设岱庙文化广场；将市值近10亿元、占地192亩的时代发展线黄金地段开辟为生态绿地健身公园。着力抓好徂汶净水厂、城市地下给排水管网、泰城垃圾焚烧发电续建工程等项目建设。开展拆迁会战攻坚，财源街、岱北街拆迁和灵山大街、青年路、望岳西路多年“老大难”问题得到解决，全市共治理违法建设9397处、623万平方米。以全国文明城市创建和国家卫生城市迎审为抓手，抓好户外广告牌匾提升、公厕改造、生活垃圾分类等工作，拆除广告牌52.8万平方米。城镇建设取得新成绩，岱岳区入选全国新型城镇化质量百强区，新泰市被评为全国十佳生态文明城市，宁阳县被列为省第三批新型城镇化试点县，大汶口镇被评为省级绿色生态示范镇；我市以全省第3名的成绩，顺利通过国家卫生城市复审，并实现国家卫生城市（县城）全覆盖；全国文明城市创建工作获得了全省7个提名城市中第2名、全国113个地级提名城市中第12名的好成绩，肥城市、新泰市在全省21个创建县中分列第1名和第4名；我市在全省新型城镇化三年综合考核中列第4位，被省政府授予“山东省适宜人居环境奖”。重大基础设施建设顺利推进，全市在建和开展前期工作的高速公路8条，实施国、省道项目17个，新改建农村公路1051公里。

（五）三大攻坚战取得新进展。扎实开展“增信贷、降不良、打逃债、树诚信”专项行动，清收不良贷款8.6亿元。2018年末，全市人民币各项存款余额3647.68亿元、比年初增长9.7%；人民币各项贷款余额2354.32亿元、比年初增长11.7%。东顺集团在香港成功并购上市。政府债务率比上年压减17.5个百分点。全力推进精准脱贫，1.22万建档立卡贫困人口全部脱贫退出；东平县易地扶贫搬迁、黄河滩区迁建、移民避险解困“三大工程”稳步实施，3.5万人实现回迁，全国水库移民工作会议在东平县召开。扎实推进泰山区域山水林田湖草生态保护修复工程，开工项目215个，完成投资46.8亿元。中央环保督察组反馈意见涉及我市的21个具体问题，8个已完成整改，13个达到序时进度。省环保督察组反馈意见涉及我市的25个具体问题，20个完成整改，5个正积极推进。全年推广洁净煤20万吨。山东宁阳生物化工高技术产业园区、肥城市循环经济产业园区、新泰市循环经济产业示范区、泰安大汶口工业园化工区等4家化工园区获省政府批准。2个土壤污染防治项目纳入中央项目储备库。全市空气质量优良天数达到207天，居全省内陆城市前列。全市482条河流、688座水库、1444座重点塘坝共设河（湖）长4534名，实现河湖水域管理全覆盖。黑臭水体治理取得了阶段性成果。全国水生态文明城市创建顺利通过验收。扎实开展“绿满泰安”行动，全市自然保护区、国家湿地公园、中国森林氧吧分别达到6个、3处和3处，有10个乡镇、55个村（居）成功创建市级以上“森林镇村”，新泰市荣获首批“山东省森林城市”称号，宁阳县被授予“全国绿化模范县”称号。

（六）民生保障水平持续提升。全市一般公共预算中民生支出达到303.4亿元，占财政支出的79.6%。加快解决“大班额”问题，新建、改扩建学校、幼儿园120所；“全面改薄”完成投资2.38亿元，超额完成任务。加快分级诊疗制度建设，县域内就诊率达到92.02%；国家基本公共卫生服务项目人均补助经费达到55元。新增就业5.25万人，城镇登记失业率2.21%。发放各项社会保险待遇180.74亿元、增长10.1%。城乡低保和农村特困供养对象93836人，累计发放救助金3.12亿元。妥善解决拖欠农民工工资问题。加强养老服务体系建设，全市新增养老床位2260张，新建城市社区老年人日间照料中心10处、农村幸福院37处。加快保障性住房建设，实施棚户区改造5.8万套，基本建成2.1万套。整治老旧住宅小区20个、91.6万平方米，修复破损玻璃19.8万平方米，开展既有住宅增设电梯试点工作。发放城镇住房保障家庭租赁补贴4092户，改造农村危房2561户。归集住房公积金48.5亿元，发放公积金贷款26.4亿元。大力繁荣文化体育事业，村级综合性文化服务中心实现全覆盖，举办各级各类体育健身赛事活动3172项，列全省第一，取得省第24届运动会金牌榜第7名好成绩。国防动员、双拥共建、人民防空、民兵预备役、军民融合和退役军人保障工作取得新进展。妇女儿童、残疾人、老龄工作都有新提高。外事侨务、民族宗教、史志档案等都有新进步。持续开展平安泰安建设，深入推进扫黑除恶专项斗争，打掉恶势力犯罪集团21个、恶势力团伙46个。圆满完成全国“两会”、上合组织青岛峰会、改革开放40周年庆祝活动等重大安保维稳工作任务。毫不放松抓好社会治理创新、安全生产、重大动植物疫病防控、食品药品安全监管等工作，妥善处置“4•17”山火、“8•19”山洪自然灾害等突发事件，社会保持和谐稳定。

（七）政府自身建设不断加强。认真学习习近平新时代中国特色社会主义思想，深入贯彻落实习近平总书记视察山东重要讲话、重要指示批示精神，自觉肩负党要管党、全面从严治党政治责任。推进“两学一做”学习教育常态化制度化，扎实开展“大学习、大调研、大改进”。严格落实中央八项规定及实施细则精神，持之以恒纠治“四风”，严厉查处各类违法违纪案件和不担当不作为问题，作风建设更严更实。大力弘扬新时代泰山“挑山工”精神，营造干事创业的浓厚氛围。严格依法行政，努力高效施政，法治政府建设扎实推进。及时解决群众合理诉求，主动接受各方面监督，认真办理人大代表建议和政协委员提案，答复率均达100%。12345政务服务热线接听群众来电52.8万件，回访29.4万人次，群众满意率达到94.42%。持续压减“三公”经费，市级财政拨款安排的“三公”经费同比下降14.6%。政务资源信息整合共享、政务公开、政风行风评议积极推进，政府公信力进一步提升。

各位代表，这些成绩来之不易。这是全市上下坚决贯彻党中央、国务院决策部署和省委、省政府工作要求的结果，是市委统揽全局、加强领导和市人大、市政协监督支持的结果，是历届老领导、老同志关心支持的结果，是全市人民群策群力、实干奋进的结果。在此，我代表市人民政府，向全市广大干部群众、各民主党派、工商联、无党派人士，向人民解放军和武警驻泰部队及驻泰单位，向关心支持泰安发展的社会各界，表示崇高的敬意和衷心的感谢！

在肯定成绩的同时，必须清醒地看到，我们的工作还存在不少困难和挑战。主要表现在：经济总量仍然偏小，实体经济发展面临多重压力，能源、环保“天花板”制约凸显，外部约束日益趋严；财政收支矛盾突出，稳增长压力较大；动能转换内生动力仍然不足，新兴产业发展快但占比小，企业创新能力不强；“双招双引”力度不够大，成效不够好；要素保障不够充分，民营和小微企业融资难融资贵问题还未得到有效缓解，防范化解金融风险压力依然较大，营商环境与市场主体期待还有较大差距；城市综合服务功能还不够完善，城市管理、社会治理短板多、堵点多，与群众要求还有较大差距；生态保护和污染防治任务依然繁重；贫困人口持续稳定脱贫的压力仍然较大；干部工作本领和工作作风与新时代新要求还不相适应，部分干部境界不高、观念不新、作风不实，行政效能、发展环境还需进一步提升和优化，等等。这些矛盾和问题，需要我们系统研究和分类解决。

二、2019年工作安排

2019年是新中国成立70周年，是决胜全面建成小康社会的关键之年，也是加快新旧动能转换、实现高质量发展的重要一年。今年政府工作的总体思路是：以习近平新时代中国特色社会主义思想为指导，认真贯彻党的十九大和十九届二中、三中全会精神，深入落实习近平总书记视察山东重要讲话、重要指示批示精神，按照中央决策部署和省市委工作要求，坚持稳中求进工作总基调，坚持新发展理念，坚持以供给侧结构性改革为主线，以新旧动能转换重大工程为引领，以“工作落实年”为总抓手，加快实施创新驱动发展和人才强市战略，大力传承和弘扬新时代泰山“挑山工”精神，强化党建统领和人才支撑，聚力推进产业发展、乡村振兴、城市规划建设管理工作实现新突破，努力建设先进制造、文化旅游、医养健康强市，打造创新创业、山水生态名城，不断塑造高质量发展新优势，促进经济持续健康发展和社会大局稳定，以优异成绩庆祝新中国成立70周年。

主要预期目标是：生产总值增长6%左右，一般公共预算收入增长5%，收入质量进一步提升；投资结构持续优化；消费对经济增长的贡献率进一步提高；城镇登记失业率控制在3.5%以内，城镇、农村居民人均可支配收入分别增长7%和7.5%左右；全面完成省下达的节能减排降碳约束性目标。

以上预期目标，突出了稳中求进工作总基调，突出了高质量发展新要求，有利于稳定市场预期，有利于缓解要素制约、提高生态承载、化解历史包袱。工作中，我们将按照中央“巩固、增强、提升、畅通”八字要求，紧密联系泰安实际，坚持走改革开放的创新之路、攻坚克难的奋斗之路、高质量发展的效益之路、绿色发展的生态之路、以人民为中心的富民强市之路，更有成效地稳增长、促改革、调结构、惠民生、防风险、保稳定，更加坚定地稳就业、稳金融、稳外贸、稳外资、稳投资、稳预期，努力实现有质量、有效益、有速度的持续稳定增长。工作中，要按照市委要求，在全市上下吹响“同心同德干事业、攻坚克难抓落实”的号角，凝聚一切力量，用好一切资源，戮力同心、和衷共济，砥砺奋进、奋勇争先，以更高标准、更大担当，加快新旧动能转换、实现高质量发展，奋力谱写泰安改革发展的新篇章。重点做到以下七个方面的“狠抓落实”：

（一）突出抓好产业高质量发展，在加快新旧动能转换上狠抓落实。坚定不移实施“产业兴市”战略，进一步强化抓产业就是抓发展、抓好高质量产业发展就是对泰安未来负责的思想，精准编制并落实好新旧动能转换十大产业专项发展规划，尽快形成一批布局结构优、规模体量大、延伸配套好、支撑带动强的千亿级产业集群。积极推动玻纤老厂区搬迁和新园区建设、晋煤明升达洁净煤气化、岱银品牌设计研发中心、石横特钢泰安建筑用钢产业集群等一批项目建设，实现传统产业动能接续提升；大力推进康平纳年产20万吨筒子纱智能染色工厂、泰开工业机器人等一批高端装备和智能制造、新一代信息技术、现代物流、新能源和节能环保产业项目建设，形成新兴产业动能新支撑。加快泰山智能制造产业研究院建设，构建研发机构、产业联盟、产业基金、产业基地、人才团队“五位一体”平台，形成产业智能制造的泰安解决方案。推动军民融合发展，抓好中航重车、泰邦生物等企业项目建设，争取更多示范项目落户泰安。

大力培植“双50强”企业，建立完善重点企业市级领导联系制度，整合全市资源要素和政策措施，在税收、用地、人才、融资等方面给予重点支持。鼓励引导企业不断加大投入、扩大规模、延伸产业链条、扩大市场份额，力争3-5年内全市过100亿元的企业达到10家，50-100亿元的企业新增6家。用足用好100亿元的重点企业财源建设基金，年内对重点企业支持不低于30亿元。充分发挥市金控集团和财金集团作用，综合运用融资租赁、融资担保等工具，帮助企业解决融资难融资贵问题。落实好企业上市挂牌奖励政策，支持龙头骨干企业和创新型企业上市融资。

加快编制现代服务业发展规划，推进先进制造业与现代服务业深度融合，带动电子商务、研发创意、大数据等产业发展壮大。推进铁路物流园区、岳华能源泰山内陆港规划建设；持续支持峰松物流无车承运人全国试点，力争全年交易量过100亿元、税收过10亿元，进而打造千亿级现代物流产业集群。立足泰安生态、科教、文化优势，瞄准市场需求，着力发展医疗服务、休闲旅游、健康养老、体育健身、研学体验等新兴产业，努力打造国际知名的“养老泰安•养生泰山”医养健康品牌。

坚定不移把文化旅游业作为战略性支柱产业全域性谋划、一体化推进。年内编制完成《泰安市文化旅游产业发展总体规划》。继续大力实施旅游服务提升“四大工程”，建设一批旅游特色街区。加快泰山新闻出版小镇等文旅重点项目建设，启动天平湖核心区景观提升工程。推动乡村旅游提档升级，加快构建由特色小镇、休闲农庄、现代农业园区、精品民宿、特色村和农家乐组成的乡村旅游体系。更加注重文旅市场主体培植和壮大。把“游客为本”理念落实到旅游每一个环节、每一个细节，开展文明执法、柔性执法，营造良好旅游环境。

加强对实体经济特别是工业经济运行的监测分析，围绕投资、财税、规上企业、进出口等主要经济指标，紧盯运输量、用电量、订单量等先行指标，科学引导和把握增长预期，确保基本面稳定。强化对重点骨干企业的运行监测，帮助解决企业在市场、资金、技术、人才、水电油运气等方面的问题，保障企业需求，助力企业发展，确保我市整体经济形势稳中有进，力争进中见好。

（二）突出抓好乡村振兴战略实施，在加快实现农业农村现代化上狠抓落实。在产业振兴上，重点培植壮大农业“新六产”，切实抓好泰山区国家级现代农业产业园、新泰省级现代农业产业园、汶阳田现代农业产业园、泰安高新区省级农业科技园等园区建设。抓好粮食生产，维护粮食安全。调整优化农业结构，做大做强农业龙头企业，加快培育泰山茶、泰山板栗、现代奶业、泰山樱桃、新泰百合、肥城佛桃、苗木花卉、东平湖产品、宁阳大枣等一批农业优质、绿色产业，努力打造过10亿元的特色产业。深入创建“省级农产品质量安全市”，省级农业标准化基地达到80个。加快推进区域公用品牌、企业品牌、产品品牌“三牌”同创，年内省级以上品牌总数达到25个。在人才振兴上，重点实施好职业农民技能提升、乡土人才培育示范、乡土人才定向培养等计划，逐步构建起市、县、乡、村四级培训网络。完善农民返乡创业政策，支持各类人才返乡下乡创新创业。在文化振兴上，重点推进新时代文明实践中心建设试点，深化乡村文明行动，传承优秀传统文化，形成积极进取、向上向善的文化氛围和价值追求。在生态振兴上，重点开展农村人居环境整治三年行动，统筹推进乡村振兴和美丽乡村、田园综合体建设，抓好垃圾污水处理、厕所革命、村容村貌提升，年内新增省级美丽乡村示范村28个，建成“四好农村路”1000公里。在组织振兴上，重点加强农村基层党组织建设，培养一批政治素质好、能带富、善治理的农村基层组织带头人。建立健全党组织领导，自治、法治、德治相结合的乡村治理体系，为乡村振兴提供组织保障。

（三）突出抓好“双招双引”和重点项目建设，在加快培育发展新动能上狠抓落实。坚持把“双招双引”作为加快新旧动能转换、发展产业经济的总抓手，积极组织开展“产业链招商、专业招商、以商招商、平台招商”四大招商行动，有目标地走出去，有重点地请进来，加大与世界500强、跨国知名企业、大型央企和百强民企的对接力度，着力引进一批领军企业、领先技术和高层次人才。加强与北邮、北航、360企业安全集团等合作，努力培植壮大网络安全产业。继续发挥泰山国际登山节招商引资大舞台、大擂台作用，压实领导责任，形成整体合力，务求“双招双引”实效。

持续强化科技人才支撑，在全省率先打造以“塔形”科技创新体系为核心、以泰山创新谷为载体的“政产学研金服用”创新创业共同体，加强与国内知名高校、专家、团队合作，年内实现10家产业技术研究院实体化运作，以企业为主体的产业技术研究院突破300家。加快培育一批“瞪羚”、隐形冠军和单项冠军企业。全面落实人才“金十条”政策，加大高端人才培养引进和使用力度。着力优化我市人力资源结构，大力实施“泰汶专才”计划，鼓励各高职院校针对“十大产业”设置专业，校企共建，培养专而有用的高技能人才。加强知识产权保护，促进发明创造和转化运用。

加快推进重点项目建设，实现重点项目管理由传统性投资向“四新四化”产业转变，动态管理市级新旧动能转换重大项目库，突出抓好6个省级重点项目、100个市级重点项目建设，年度计划完成投资300亿元以上，重点推动泰安二期抽水蓄能电站、泰山文旅健身中心、泰山Office、泰山玻纤新生产线、华能光纤预制棒和光纤制造一期、泰山•桃花源、振挺精工智能化生产线、中信重工开诚智能特种机器人等项目取得新进展，争取更多项目列入省新旧动能转换优选项目库。加大基础设施建设力度，推进实施泰安汽车总站改造，切实抓好济泰高速、青兰高速、京台高速泰安段拓宽等在建工程，扎实推进泰东高速、聊泰铁路、莲花山机场改扩建等项目前期工作。

为确保重要工作重大工程重点任务落实落地见效，市委、市政府确定实行“5+2”工作模式，即成立产业经济发展、城市规划建设管理、文化旅游产业、乡村振兴、党建和人才5个重点任务工作小组，成立泰山区域山水林田湖草生态保护修复工程城区项目和农村项目2个指挥部，由市重点工作小组协调推进办公室一体推进、一体调度、一体考核，挂图作战、标旗推进。各工作小组和指挥部要各司其职，攻坚克难，确保“双招双引”工作实现大的突破，确保重点项目重点工程按计划顺利实施。

（四）突出抓好规划建设管理，在提升城市功能和补短板、疏堵点上狠抓落实。泰城是老百姓的泰城。城市规划建设管理要更加强化以人为本理念，更加突出坚持以市民为中心，重点在解决好补城市短板、疏市民堵点上狠下功夫。在城市规划上，以高度的历史责任感，重点抓好省委、省政府批复的《泰安市城乡一体空间发展战略规划》的实施工作，根据泰城管辖权调整实际，实现控制性详细规划全覆盖，切实控制好“山体线”“水岸线”“天际线”。强化规划刚性约束，认真抓好专项规划编制实施，加强规划执法管理，进一步改变规划围着项目转的状况。在城市建设上，重点在打通出口、打通连接线、打通城市动脉上下功夫，推进灵山大街东段、泰楼路、泰山大街、北上高大街、龙潭路（老电校段）改造等道路建设，解决好“断头路”“盲肠路”“丁字路”问题。打通背街小巷、完善微循环、构建街区式道路网络。规划好城市道路“三块板”，合理划分快车道、慢车道、人行道，优化城市道路红线、绿线和建筑后退控制线。加快推进天然气储气调峰项目、国电机组高背压改造、东部供热主管道和西部热源厂规划建设，积极发展地源热泵、空气源热泵、电热采暖等新型供热方式。加快做好范镇LNG项目复产工作和徂汶净水厂建设。系统提升城市绿化水平，做到全城有定位、分路有主题、一街一风景、一景一记忆。持续开展城市绿荫行动，按照“300米见绿、500米见园”标准，提高城市公园绿地服务半径覆盖率。加快老城区改造提升步伐，年内启动旧村改造项目17个。完善房地产开发管理机制，做好市场规范、宏观控制工作。切实提高老旧小区物业管理水平，着力解决好居民反映强烈的道路不平、路灯不亮、供气不暖、下水不通和宠物管理混乱等方面的问题。积极推进既有住宅增设电梯试点工作。在城市管理上，积极推进智慧城市建设，重点抓好“智慧城管”平台管理应用，按照全国“示范城市”标准，全力提升市政管养水平。大力推进生活垃圾分类，持续开展城市违法建设治理，推进城乡环境综合整治和旅游秩序整治，巩固国家卫生城市复审成果，继续攻坚全国文明城市创建工作。积极推进新型城镇化建设，尽快实现城镇基本公共服务全覆盖。

（五）突出抓好三大攻坚战，在形成常态化良性机制上狠抓落实。坚持底线思维，坚决打好防范化解重大风险攻坚战。加强地方金融监督管理，持续深入开展“增信贷、降不良、打逃债、树诚信”专项行动，加快构建全域金融风险防控体系。加快推进金融商务区建设，积极争取全省绿色金融试验区。有序处置地方政府存量债务，规范政府举债融资行为。严格落实党政领导干部安全生产责任制，完善“安如泰山”科学预防体系，启动安全发展示范城市建设。深入开展化工产业安全生产转型升级专项行动，加快企业进区入园步伐。加强对道路交通、煤矿和非煤矿山、建筑工地等重点领域专项治理。以“四个不能省”的要求，确保全市安全生产形势持续平稳。大力实施餐饮质量安全提升工程和药械质量安全护航行动。创新社会治理，做实网格化服务管理。深入推进平安泰安建设，持续开展扫黑除恶专项斗争。推进公共法律服务体系建设，深化普法宣传教育。健全社会信用体系，完善失信联合惩戒机制。加强和改进信访工作。加强应急管理和处置能力建设，确保社会和谐稳定。

坚持为民情怀，坚决打好精准脱贫攻坚战。把防止返贫放在更加重要的位置，紧盯重点贫困区域、特殊贫困群体两大重点，着力解决影响“两不愁三保障”的突出问题。统筹抓好东平县易地扶贫搬迁、黄河滩区迁建、移民避险解困“三大工程”。推进脱贫攻坚与乡村振兴战略相衔接，着力提高贫困村、贫困群众的内生动力和自我发展能力。开展脱贫攻坚“回头看”，严查扶贫领域腐败和作风问题，确保扶贫工作务实、脱贫过程扎实、脱贫结果真实。

坚持标本兼治，坚决打好污染防治攻坚战。认真抓好中央、省环保督察及“回头看”反馈意见整改，全面落实环保行动方案，压实生态环境责任。实施“七大专项治理行动”，坚决打好“七大标志性攻坚战”，全面完成国家、省下达的总量减排、环境改善指标任务。落实“四减四增”三年行动方案，统筹抓好工业污染源、移动污染源和农村秸秆焚烧、烟花爆竹等治理工作，确保空气质量优良天数持续增加，坚决打赢蓝天保卫战。

山水林田湖草是一个生命共同体。实施好山水林田湖草生态保护修复工程，是践行习近平生态文明思想的重要内容，是省委、省政府交给我们的重大政治任务，也是泰安高质量绿色发展的重大机遇。该工程总投资166亿元，包含67个项目、326个子项目，要求2020年6月底前全面完成。目前完成投资占总投资的28.1%，任务艰巨、难度很大，必须突出重点，聚力攻坚，今年确保完成工程总量的70%。突出水环境治理，加快实施牟汶河、柴汶河、康汇河流域以及环东平湖水环境综合治理工程；加快推进泰城明堂河、梳洗河、芝田河、泮河等7条河流生态修复和综合整治，建设沿河生态景观带；着力抓好城区污水管网、沿大汶河湿地、污水处理厂建设改造、胜利渠等重点河道清淤和城区雨污分流等工程。加大破损山体修复力度，将山体治理与土地整理结合起来，把破损山体修复与工矿废弃地复垦、工业区建设及居民生活条件改善结合起来，统筹谋划、一体推进；加大泰山石保护力度，坚决实行封山禁采、封场禁售，关闭取缔大型石头存放和交易场所。针对农业面源污染问题，切实抓好土地整治和高效生态农业治理。科学编制全市生态文明建设规划，持续推进“绿满泰安”行动，加大自然湿地和新建湿地保护力度，努力将泰山大生态带打造成“山青、水绿、林郁、田沃、湖美、草丰”的生命共同体。要强化工程项目推进，抓住雨季之前施工黄金期，倒排工期，加快建设。强化工程资金保障，通过PPP模式筹集资金60亿元，落实专项基金60亿元。强化工程质量效益，发挥特聘专家的作用，加强工程项目监理，确保建成一批特色工程、示范工程、优质工程，创出“泰安经验”，向省委、省政府和全市人民交一份满意的答卷。

（六）突出抓好改革开放，在激发经济社会发展活力上狠抓落实。全面完成市县机构改革，不断提高政府效能。强化开发区改革开放排头兵、新旧动能转换主战场作用，更加突出经济发展功能，实行“管委会+公司”运行机制，推进用人制度和薪酬制度改革，实现各类园区的多元化投入、企业化管理、市场化运作和专业化服务。积极推进预算管理改革，全面实施预算绩效管理，加快建立现代财政制度。坚定不移落实好减税降费政策，让企业获得更大的自我发展能力。稳妥推进农村改革，年内全面完成农村集体产权制度改革任务。深化国资国企改革，积极发展国有资本、民营资本交叉持股的混合所有制经济。持续深化“放管服”改革，做到审批至简、服务至善，突出抓好企业开办、不动产登记、建设项目审批三个领域，实行全程代办、高效贴心服务，切实打造优质政务服务品牌。落实好扶持民营经济等有关政策，鼓励支持民营企业建立现代企业制度，加快技术、管理、产品等一系列创新。更加重视企业家队伍建设，特别是针对骨干企业和换代企业，对其管理团队和领航接力人才进行重点培养。构建亲清新型政商关系，健全政企沟通机制，提供更好服务。扩大对外开放，年内新增进出口获权企业100家以上，新增有进出口实绩企业100家以上。积极利用省举办的香港山东周、韩国商务周及中国国际进口博览会等高端平台，推动利用外资实现新突破。深入对接“一带一路”，引导更多企业积极“走出去”。

（七）突出抓好民生和社会事业，在切实提升群众获得感幸福感上狠抓落实。人民群众的获得感幸福感，就是人民政府的使命感责任感。认真抓好民生实事。市委、市政府确定了今年为民要办10件实事，要想尽千方百计把实事办实、好事办好。①城乡居民基本医疗保障提升工程，居民医保财政补助标准提高到520元，居民大病保险筹资标准提高到70元，全面落实建档立卡贫困人口、特困人员、低保对象、重度残疾人等困难人员的参保补贴和大病保险倾斜政策。②农村饮水安全保障工程，解决143个无公共供水设施村、534个水质有风险村的饮水安全问题；完成5个氟超标村改水工作；新建规模化供水工程4处。③农村敬老院“四改一定”工程，全面改善81处农村敬老院卫生间、厨房及餐厅、医疗服务、文体娱乐设施，定期组织老人外出旅游观光，打造农村特困供养人员“幸福之家”。④全市棚户区改造工程，新开工棚户区改造安置住房13396套。⑤城镇小区配套幼儿园综合整治提升工程，新建、扩建幼儿园80处，整改幼儿园14处。⑥城乡道路建设提升工程，拆迁建设好擂鼓石大街中段、环山路东延、天平北街主干道，改造建设学院路、长城西路次干道；年内确保1000个行政村实现道路“三通”（村村通、巷巷通、户户通）。⑦乡镇卫生院（社区卫生服务中心）建设工程，实施“泰山基层名医”人才工程，重点培植20个“泰安市基层医疗卫生机构特色科室建设单位”。⑧泰城老旧小区改造提升工程，年内完成堰岭小区、三联小区等近60万平方米的改造提升工作。⑨泰城农贸市场改造提升工程，年内完成岱北市场等28处农贸市场（农超）的迁建改造提升工作。⑩高铁泰安站北配楼改造工程，总改造面积9600平方米，年内基本完成候车室改造。

更加重视就业和社会保障。扎实做好高校毕业生、退役军人、农民工等重点群体就业工作，实施职业技能提升行动，确保城镇新增就业4万人。完善社会保障体系，逐步提高职工养老保险统筹层次。适时调整机关事业单位和企业退休人员养老金。完善提升居民养老保险待遇，对65周岁以上参保居民予以倾斜，居民基础养老金最低标准每5年至少调整一次。进一步提高城乡低保、特困供养、临时救助和专项救助标准，确保农村低保标准高于动态调整的省定脱贫标准。大力发展养老特别是社区养老服务业，年内新增养老床位2000张，打造10处示范性城市社区老年人日间照料中心，泰山社会福利中心确保规范运营。

优先发展教育事业。坚持立德树人，大力推进义务教育优质均衡发展，力争岱岳区、新泰市、肥城市通过国家首批评估认定。加快冯玉祥小学、市直机关幼儿园等项目建设，全年新建、改扩建中小学19所，有效解决大班额问题。切实保障进城务工人员随迁子女平等接受义务教育，做好家庭经济困难学生资助工作。完善现代职业教育体系，支持泰山职业技术学院创建省优质高职院校，支持各职业院校走产业化自我发展之路。鼓励社会力量多种形式兴办教育。支持驻泰高校做大做强，进一步发挥驻泰高校对地方发展的引领支撑作用。

大力发展卫生健康事业。加快建立现代医院管理制度和分级诊疗制度。全面推进公立医院综合改革，力争泰安市立医院建成运营，市中心医院高新区新院区加快建设。加强医疗保障工作，把更多救命救急的好药纳入医保范围，减轻群众就医负担。提升残疾预防和康复水平，推进国家康复辅助器具产业综合创新试点工作。充分发挥我市中医药特色优势，打造具有较大影响力的中医药事业和产业发展品牌。

繁荣发展其他社会事业。加快编制实施泰山文化保护传承发展总体规划，深入挖掘泰山文化和泰山精神内涵。加快泰山“挑山工”党性教育基地建设，大力弘扬新时代泰山“挑山工”精神。深入实施文化惠民工程，持续开展“送戏下乡”演出和全民阅读活动，建设“书香泰安”。加大文物保护力度，全面提高文物安全防范能力。加快岱庙文化广场建设。加强网信工作，加快建设市县融媒体。繁荣文艺创作，发展新闻出版、广播影视和档案事业。大力发展体育事业和体育产业，全力推进新体育中心建设。高度重视统计工作，认真做好全国第四次经济普查。依法管理宗教事务，增进民族团结。加强国防教育、国防动员和人民防空建设。认真做好军队退役人员安置、管理服务和双拥工作，巩固军政军民团结。关心支持老龄、妇女儿童事业发展。

三、建设新时代人民满意政府

新时代、新形势、新任务，对政府自身建设提出了新的更高要求，全市政府系统一定要坚持党的绝对领导，进一步加强法治政府、服务政府和廉洁政府建设，同心同德干事业、攻坚克难抓落实，努力建设人民满意政府。

（一）切实提高政治站位。把旗帜鲜明讲政治摆在首位，深入贯彻落实党的十九大精神和习近平总书记视察山东重要讲话、重要指示批示精神，自觉用习近平新时代中国特色社会主义思想武装头脑、指导实践、推动工作。牢固树立“四个意识”，坚定“四个自信”，坚决做到“两个维护”，不折不扣贯彻落实中央决策部署和省市委工作要求。政府系统要不断加强党性修养，努力增强政治自觉，激励广大党员干部成为忠诚、干净、担当的楷模。

（二）切实做到依法行政。深入推进依法治市，严格遵守宪法和法律，把政府活动全面纳入法制轨道。认真执行人大及其常委会各项决议决定，自觉接受人大工作监督、法律监督和政协民主监督，主动接受社会和舆论监督，认真做好人大代表建议、政协委员提案办理工作。坚持科学民主依法决策，认真听取社会各界、专家学者和社会公众的意见建议，密切与各民主党派、无党派人士和工会、共青团、妇联等群团组织的联系沟通。深化行政执法体系建设，全面落实执法责任制和问责制。

（三）切实转变工作作风。认真贯彻省市“担当作为、狠抓落实”工作部署，抓好“埋头苦干、争创一流，勇做新时代泰山‘挑山工’”主题实践活动，做到 “挑担不怕难、上山不怕险、坦途不歇脚、重压不歇肩”，努力做到咬定目标抓落实、踏实苦干抓落实、攻坚克难抓落实、勇于担责抓落实。坚持以实绩论英雄，比实干赛实绩，积极开展“七赛七比”活动，面向全国全省寻标对标竞标，省内学威海，省外学嘉兴，坚决啃下制约泰安发展的“硬骨头”，通过“四个擂台”，跑好泰安高质量发展的“接力赛”。实行“写实考核”，健全完善正向激励、容错纠错和尽职免责等工作机制，为敢担当、勇担当，能干事、干成事的干部搭建舞台、撑腰鼓劲。坚决反对和整治一切形式主义和官僚主义，采取“四不两直”开展调查研究；大力整治文山会海，文件会议减少1/3以上，各类督查考核减少50%以上。

（四）切实从严廉洁自律。全面落实从严治党主体责任，坚决履行党风廉政建设“一岗双责”。认真执行党内政治生活准则和党内监督条例，严格落实中央八项规定及实施细则精神。紧盯重大工程、重点领域、关键岗位，强化对权力集中、资金密集、资源富集部门和行业的监督，保持高压反腐态势。突出新旧动能转换、脱贫攻坚、生态环保等重点领域，推进公共资金、国有资产、国有资源和领导干部经济责任审计全覆盖。强化过“紧日子”思想，严肃财经纪律，厉行节约，勤俭办事。每一位政府工作人员，都要始终把纪律和规矩挺在前面，坚定理想信念，强化为民宗旨，做廉洁自律的表率，做真抓实干的表率，真正树立起政府勤政为民的良好形象。

各位代表，志不求易者成，事不避难者进。山东正处于“走在前列、全面开创”的关键时期，泰安正处于转型发展、爬坡过坎的关键时期，我们肩负着新时代赋予的重大责任和光荣使命。让我们更加紧密地团结在以习近平同志为核心的党中央周围，在习近平新时代中国特色社会主义思想指引下，在中共山东省委和泰安市委的坚强领导下，敢于担当，勇于创新，善于实干，乐于奉献，“同心同德干事业、攻坚克难抓落实”，为建设泰安更加美好的明天而努力奋斗！
